# Solar irradiance dictates settlement timing and intensity of marine mussels

**DOI:** 10.1038/srep29405

**Published:** 2016-07-07

**Authors:** Isabel Fuentes-Santos, Uxío Labarta, X. Antón Álvarez-Salgado, Mª José Fernández-Reiriz

**Affiliations:** 1 Consejo Superior de Investigaciones Científicas (CSIC), Instituto de Investigaciones Marinas (IIM), C/Eduardo Cabello 6, 36208 Vigo, Spain

## Abstract

Identifying the environmental factors driving larval settlement processes is crucial to understand the population dynamics of marine invertebrates. This work aims to go a step ahead and predict larval presence and intensity. For this purpose we consider the influence of solar irradiance, wind regime and continental runoff on the settlement processes. For the first time, we conducted a 5-years weekly monitoring of *Mytilus galloprovincialis* settlement on artificial suspended substrates, which allowed us to search for interannual variability in the settlement patterns. Comparison between the seasonal pattern of larval settlement and solar irradiance, as well as the well-known effect of solar irradiance on water temperature and food availability, suggest that solar irradiance indirectly influences the settlement process, and support the use of this meteorological variable to predict settlement occurrence. Our results show that solar irradiance allows predicting the beginning and end of the settlement cycle a month in advance: Particularly we have observed that solar irradiance during late winter indirectly drives the timing and intensity of the settlement onset, Finally, a functional generalise additive model, which considers the influence of solar irradiance and continental runoff on the settlement process, provides an accurate prediction of settlement intensity a fortnight in advance.

The life cycle of most benthic marine invertebrates involves a pelagic larval phase that can last for several weeks until individuals are competent to settle and a post-larval sedentary stage^1^,^2^. Larval settlement, the process that links larval and benthic stages, is determined by larval production, pre-settlement survival and transport and plays a pivotal role on the population dynamics of these animals. Many studies have addressed the effects of hydrodynamics on larval dispersal and, consequently, on the spatial variability of larval settlement^3^,^4^,^5^, while the temporal pattern of larval settlement has been mainly attributed to their reproductive cycle^5^,^6^.

The evolution of marine benthic invertebrates has resulted in synchronized reproductive cycles to produce larvae under optimal conditions for their survival^7^. Decoupling between spawning and optimal food conditions may reduce growth rates increasing the length of the larval phase and, consequently, mortality risks, such as predation and transport to unsuitable places^8^,^9^,^10^,^11^. These interactions justify the need for studies linking environmental and biological cycles to determine which factors influence reproductive timing and settlement intensity, and to understand how environmental changes may affect population dynamics.

The large-scale variability in the seasonal cycle of mussel and oyster larvae occurrence along the European coastline has been attributed to the spatial variability of water temperature and food concentration^12^. In addition, several studies have suggested long-term shifts in the reproductive cycles of marine invertebrates in response to climate change^10^,^12^,^13^. Considering the effect of solar irradiance on water temperature^14^ and food availability^15^,^16^, recent works have analysed whether solar irradiance influences the reproductive schedule of Mediterranean and Caribbean corals^17^,^18^ and the timing of the minimum meat content in adult mussels (unpublished data), which is an indicator of spring spawning events^5^,^19^.

Mussels are dominant organisms on many rocky shores worldwide, where they play an important ecological role as habitat or prey for a multitude of organisms^20^, and in the pelagic-benthic coupling^21^,^22^,^23^,^24^. In addition, mussel culture has important commercial value^25^,^26^. The Galician Rías (NW Iberian coast), located in the northern boundary of the Iberian-Canary Current upwelling system, are characterized by a high mussel production. The importance of larval production and recruitment for the management of mussel culture in this area has motivated an increasing interest on the settlement processes of mussels and the underlying environmental factors^5^,^6^,^27^,^28^,^29^,^30^,^31^,^32^.

Larval settlement in the Galician Rías is concentrated during spring-summer, in the form of successive spawning events during the upwelling favourable season^5^,^33^. Local variability in settlement intensity but a common seasonal pattern for settlement episodes has been found^29^. Furthermore, a model that explained the effects of the wind-driven upwelling regime and continental runoff on larval development, dispersal and settlement has already been published^5^. Although the effects of environmental factors such as tidal dynamics or lunar cycles on the reproductive cycle of marine invertebrates have been widely analysed, to the best of our knowledge, interannual changes in the timing of larval occurrence have been addressed neither for mussels nor for other marine invertebrates anywhere.

The two models developed up to date to fit the settlement period of marine invertebrates^5^,^17^ are based on fortnight or monthly mean values of the environmental drivers. This work takes advantage of the recently developed Functional Data Analysis (FDA)^34^,^35^, where each data is a curve recording the behaviour of a given variable along a temporal interval, to estimate the settlement period and intensity of mussels using all the information provided by the continuous monitoring of the environmental factors.

The main aim of this work is to forecast the period and intensity of mussel larval settlement on basis of meteorological variables. Taking into account the recent findings about the relationship between solar irradiance and the reproductive schedule of marine invertebrates, we will test here whether solar irradiance influences larval settlement. We also consider the wind regime and continental run-off as additional explanatory variables, given that their effect on mussel larval settlement in the study area has already been demonstrated. The predictive models were developed in two stages: we first applied Generalized Additive Models (GAM) to fit the relationship between larval occurrence and environmental factors, and then we used GAM and Functional Generalized Kernel Additive Models (FGKAM) to predict settlement intensity. Model checking and validation was conducted on the data provided by an intensive monitoring program, which measures larval settlement weekly during 5 years in the Ría de Ares-Betanzos (NW Spain, Fig. 1).

## Results

### Descriptive analysis

#### Environmental factors

Solar irradiance (*R*) exhibits a well-defined seasonal pattern (Figures S1–S6). The GAM fit (1), deviance explained = 83%) did not find interannual variability in *R* but detected a different seasonal pattern in 2009 and 2012 (Table S1), when solar irradiances in early spring (Fig. 2, left) higher than the remaining years were recorded.

The joint discharges of rivers Eume and Mandeo (Figures S1–S6) followed a seasonal pattern with high continental runoff (*Q*) in winter and low in summer. The GAM fit (deviance explained = 86.7%) found that 2012 and 2013 records were significantly lower and higher, respectively, than the rest of years under study (Table S1). Figure 2 (centre), and Table S1 show significant interannual variability, being the dry late winters in 2009 and 2012, and the high discharges recorded during the summer of 2010 the main differential features. The predominant direction of shelf winds in our study area was along the NE-SW axis during the five years (Figure S1, bottom). Wind speed (*W*, Figures S1–S6) exhibited low seasonal variability. Despite the poor fit (deviance explained = 26.7%) we found that wind speed in 2010 was significantly higher, and 2012 experienced stronger winds in late autumn than the rest of years (Fig. 2 right, Table S1).

#### Larval settlement

Larval settlement occurred between mid-spring and early-autumn (Figures S2–S6). Comparison between years identified different settlement cycles: 2009, 2012 and 2014 showed a longer settlement period, with a large peak in mid-spring followed by subsequent settlement episodes up to mid-autumn, while in 2010 and 2013 we do not observe the spring peak and larval settlement was concentrated between June and October. We only observed slight differences between 1 and 6 m in the settlement patterns (Figures S2–S6), which were not significant by nonparametric covariance analysis (p-values > 0.1). Therefore we focus on larval settlement at 1 m hereafter.

### Modelling the probability of larval settlement

We have considered four candidate models to estimate the probability of larval settlement (see Methods). We first fitted larval occurrence according to the average solar irradiance recorded 30–45 days prior to sampling (*Y* ~ *R* (2)). Then, we incorporated the effects of the average continental runoff recorded 10–30 days before sampling (*Y* ~ *R* + *Q* (3), where + denotes additive effects), mean wind speed (*W*) and direction (*θ*) recorded 10–30 days before sampling (*Y* ~ *R* + W*θ (4), where + denotes additive effects, and * interaction), and we finally considered the effects of the three environmental factors on larval occurrence (*Y* ~ R*Q + W*θ (5)).

Even for the simplest model, which estimates larval presence from the mean solar irradiance recorded 30–45 days before sampling, the probability of correct classification in the training sample was above 80% for absence and above 90% for presence of larvae (Table 1, Figure S7). Comparisons between models *Y* ~ *R* (2) and *Y* ~ *R* + *Q* (3) indicates that including the mean continental runoff recorded 10–30 days before sampling did not provide significant gains. Model *Y* ~ *R* + W*θ (4), which incorporates the mean wind regime from the previous 10–30 days to models *Y* ~ *R* (2), and *Y* ~ R*Q + W*θ (5), which takes into account solar irradiance, wind regime and continental runoff, provided higher probabilities of correct classification than model *Y* ~ *R* (2) for both the training and validation samples, though differences between models were small. The Wilcoxon test (Table 2) indicates that the most complex approach, model (5), outperformed the others in the training sample. In the validation sample model *Y* ~ *R* + W*θ was significantly better than models *Y* ~ *R* and *Y* ~ *R* + *Q*, but we did not observe any significant gain incorporating continental runoff to model *Y* ~ *R* + W*θ.

Comparison between the performance of models *Y* ~ *R* (2) and *Y* ~ *R* + W*θ (4) (Fig. 3, Table S2) shows that model *Y* ~ *R* + W*θ provided higher probabilities of correct classification than *Y* ~ *R* when fitted over the whole period. However, we observe a balance between type I and type II errors for cross-validated predictions (see 2010, 2014 in Table S2) or even a better performance of the simplest model (see 2012 in Table S2). It should be noted (see Fig. 3, right) that misclassifications were detected at the onset or end of the settlement season, corresponding to weeks with settlement intensities close to the threshold (500 ind/m) and fitted probabilities close to 0.5.

Both models identified the settlement season in 2014 from April to November (Fig. 3, left), although model *Y* ~ *R* + W*θ (4) detected the onset of the season a week earlier and the end of the settlement period a week later than model *Y* ~ *R* (2). These results suggest using a GAM with the fortnightly mean of solar irradiance to estimate the probability of larval settlement. This approach is more cost-effective than model *Y* ~ *R* + W*θ, which also requires the monitoring of wind regime, and predicts larval settlement a month earlier, while the prediction lag for *Y* ~ *R* + W*θ reduces to 10 days. Furthermore, model *Y* ~ *R* provides an irradiance threshold (*R* = 11.46 MJ/(m^2^day)) for larval occurrence.

### Modelling settlement intensity

Two-stage GAM and FGKAM (functional generalized kernel additive models) were applied to estimate settlement abundance (see Methods). In the two-stage GAM fits we used solar irradiance to fit larval presence, and considered solar irradiance and continental runoff (*N* ~ *R* + *Q* (6)), and solar irradiance, continental runoff and wind regime (*N* ~ R*Q + W*θ (7)) as explanatory variables for settlement intensity (*N*). The FGKAM estimated settlement intensity from curves recording the values and variability of the environmental factors during a month (45 to 15 days prior to sampling), we first fitted *N* according with solar irradiance (*N* ~ *fR* (8)), and then incorporated the effect of continental runoff (*N* ~ *fR* + *fQ* (9)).

The outliers observed in the MSE and MAE of model *N* ~ R*Q + W*θ, which considered the joint effect of 1 month lagged mean solar irradiance, and 10 days lagged mean continental runoff and wind regime, indicate that this model is a bad candidate (Figure S8, left). We do not observe differences between the remaining models in terms of MSE, but the FGKAM with solar irradiance and continental runoff as explanatory variables (9) showed a better performance in terms of MAE (Figure S8, right) than the GAM depending on the same environmental factors (6) and the FGKAM based on solar irradiance (8).

In view of these results, we compared the fits provided by the generalized additive and functional models that estimate settlement intensity according to the mean values (*N* ~ *R* + *Q*) and curves (*N* ~ *fR* + *fQ*) of solar irradiance and continental runoff. Comparison between their residuals, MSE_(9)_/MSE_(6)_ = 0.66, and MAE_(9)_/MAE_(6)_ = 0.59, indicates that curves recording the behaviour of solar irradiance and continental runoff over a month (*N* ~ *fR* + *fQ*) led to a better fit of settlement intensity than mean values of the same environmental factors (*N* ~ *R* + *Q*). Figure 4 confirmed the goodness-of-fit of the functional model (*N* ~ *fR* + *fQ*). This model was able to reproduce the different settlement cycles recorded along the study period, although it underestimated some spring peaks.

## Discussion

Larval settlement of marine invertebrates is driven by larval production, pre-settlement growth and survival, and larval transport. Up to date, this process has been mainly linked with hydrodynamics as driver for larval dispersal and the subsequent spatial variability of larval settlement^3^,^4^,^5^. The temporal pattern of larval settlement has been mainly attributed to the reproductive cycle of marine invertebrates. Identifying the environmental factors underlying interannual variability of the onset, extension and intensity of larval settlement is crucial to a better understanding of this process. This work aims to provide cost-effective models to predict the settlement period and intensity of the mussel *M. galloprovincialis* in the temperate NW Iberian coast according with meteorological factors.

Most studies dealing with the reproductive cycle of marine invertebrates have identified water temperature and food availability as the main factors driving the timing of reproduction^10^,^13^. Although in coastal areas sea water temperature is modulated by continental runoff and the wind regime at the short-term scale, sea water temperature is ultimately driven by solar irradiance, and the specific heat capacity of water leads to 1–2 month delay in the response of water temperature to changes in solar irradiance^14^,^18^,^36^. These relationships suggest that solar irradiance may have an indirect delayed influence on the settlement dynamics of marine invertebrates^17^,^18^. On the other hand, several studies have determined that the hydrographic and wind regimes determine larval dispersal and settlement of *Mytilus spp.* and other marine benthic invertebrates^4^,^20^,^37^,^38^,^39^,^40^,^41^.

In the particular case of the Ría de Ares Betanzos, late-winter solar irradiance has been found to drive the spring spawning onset of mussels, seston quality is mainly driven by the joint effects of wind regime and continental runoff^42^, and the upwelling regime affects larval transport, survival and settlement^5^. These considerations support the use of solar irradiance, wind regime and continental runoff as explanatory variables to develop cost-effective models for larval settlement.

This work identified interannual variability in the settlement cycle of *M. galloprovincialis*: there were years with a prolonged settlement period, with a high peak in mid-spring followed by subsequent settlement episodes up to mid-autumn (2009, 2012, 2014), and years characterized by a shorter settlement season concentrated between June and October (2010, 2013). Thus, in addition to the long-term shift from one to two spawning periods in response to climate change found in recent studies^10^,^12^,^13^, our results suggest short-term variability in the reproductive cycle of mussels at temperate latitudes in response to interannual shifts in the environmental conditions. The contrasting results obtained for the Ría de Ares-Betanzos, where some studies identified a single spawning event in summer^19^, while others found a large spring peak followed by subsequent events up to early autumn^5^, are in agreement with the interannual changes found in this work.

We have seen that the fortnight mean solar irradiance is a good predictor of the probability of larval settlement a month ahead, which allows us to predict the onset and end of the settlement period. Apart from predicting the settlement period, this model provides insight into the influence of solar irradiance on the reproductive cycle of mussels. Our results suggest that the phenology of mussel spawning is mainly driven by the time when solar irradiance reaches a certain threshold (*R* ≈ 12 MJ/(m^2^day)). Thus, late winters with high solar irradiances promote earlier spring spawning events and the subsequent spring settlement peak, while late winters with low solar irradiances delay the onset of the reproductive and settlement cycles. These results are in agreement with the strong correlation found between the mean solar irradiance during February and the phenology of the minimum meat content of adult mussels, which suggested that high solar irradiances during February may enhance early-spring spawning events.

We have seen that a functional GAM fit using monthly curves of solar irradiance and continental runoff to estimate settlement intensity outperforms the GAM fit based on mean values of the same environmental factors. This result highlights the advantages of using functional data, which provide a valuable tool to incorporate all the information provided by continuous monitoring of meteorological and environmental factors in biological models. This information is particularly valuable in our case because, as explained above, the effects of solar irradiance and continental runoff on seawater temperature and food availability, which drive larval production and survival, are not immediate. Furthermore, as the larval pelagic phase of *M. galloprovincialis* lasts between 10 and 30 days^2^,^6^ the curves of solar irradiance and continental runoff from 15 to 45 days prior to sampling allowed us to include in the model the influence of these factors on larval production, larval growth, and pre-settlement survival^29^.

In conclusion, this work found that the settlement cycle of *M. galloprovincialis* at intermediate latitudes may experience interannual shifts in response to short-term environmental changes. These results confirmed the important role of solar irradiance on the settlement cycle of mussels. In particular solar irradiance during late winter indirectly drives the timing and intensity of the settlement onset. Finally, taking advantage of the effects of solar irradiance and continental runoff on water temperature and food availability, which have been recognized as important factors for larval supply, survival and growth^10^,^12^,^13^, we have developed two cost-effective models to predict the onset and end of the settlement season, and settlement intensity during this period. These models may provide valuable information for the development of ecological and production management strategies.

## Methods

### Larval settlement

**Figure 1:**
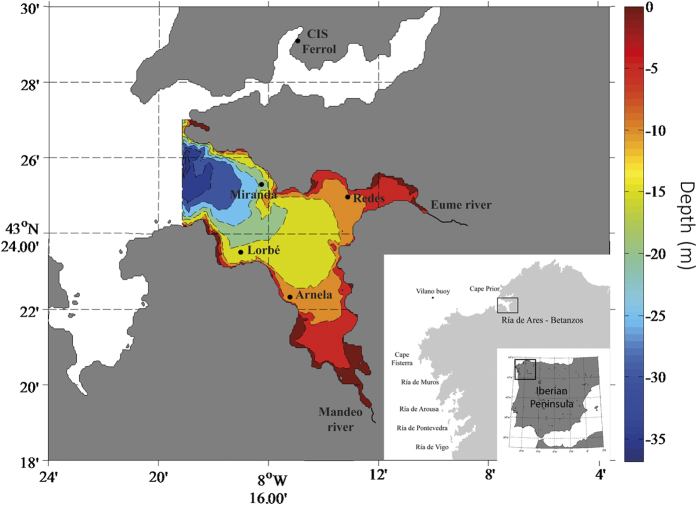
Bathymetry of the Ría de Ares-Betanzos (NW Iberian coast). Settlement monitoring was conducted in Arnela, located in the inner-southern shore. Rivers Eume and Mandeo (continental runoff), Vilano Buoy (wind regime), and CIS-Ferrol meteorological station (solar irradiance). These maps were prepared with Matlab. version: MATLAB R2009b. URL: www.mathworks.com.

**Figure 2:**
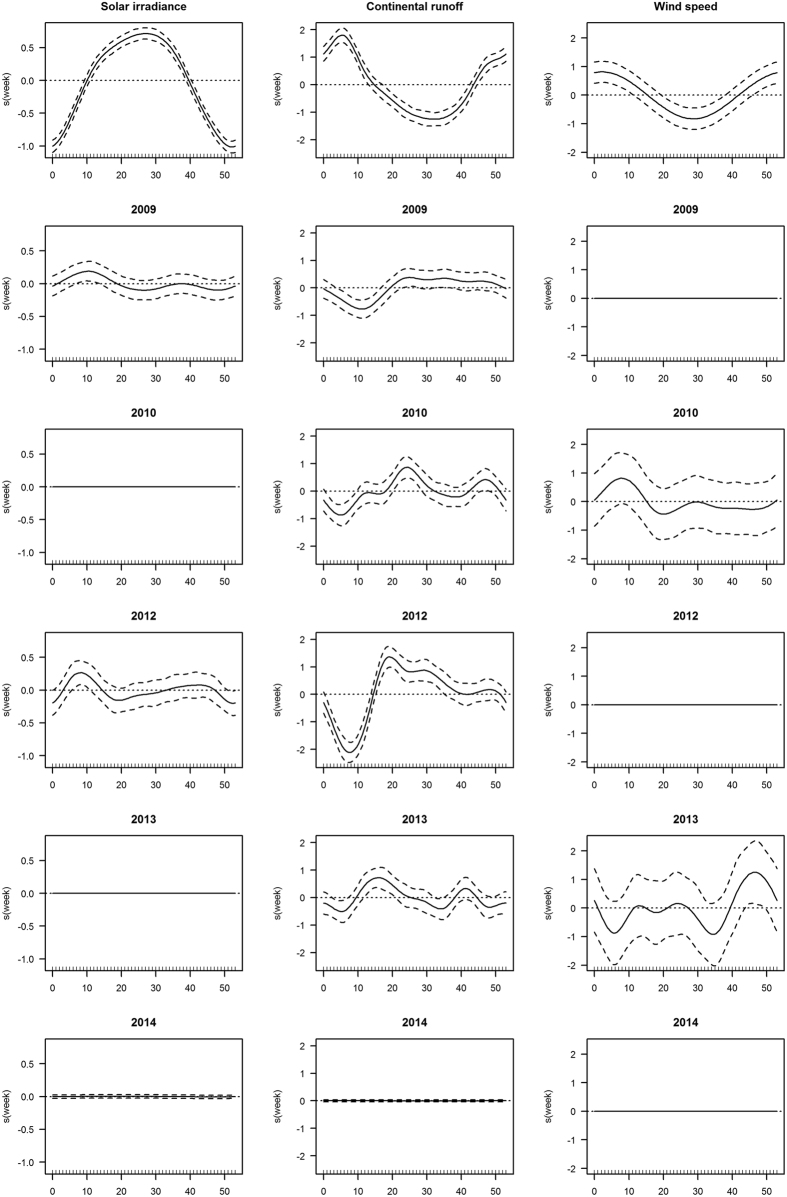
Smooth terms of the GAM fits that determine the mean seasonal patterns and interannual variability of solar irradiance (left, Gamma family and logarithmic link), continental runoff (centre, Gamma family and logarithmic link), and wind speed (right, Gaussian family and identity link).

**Figure 3:**
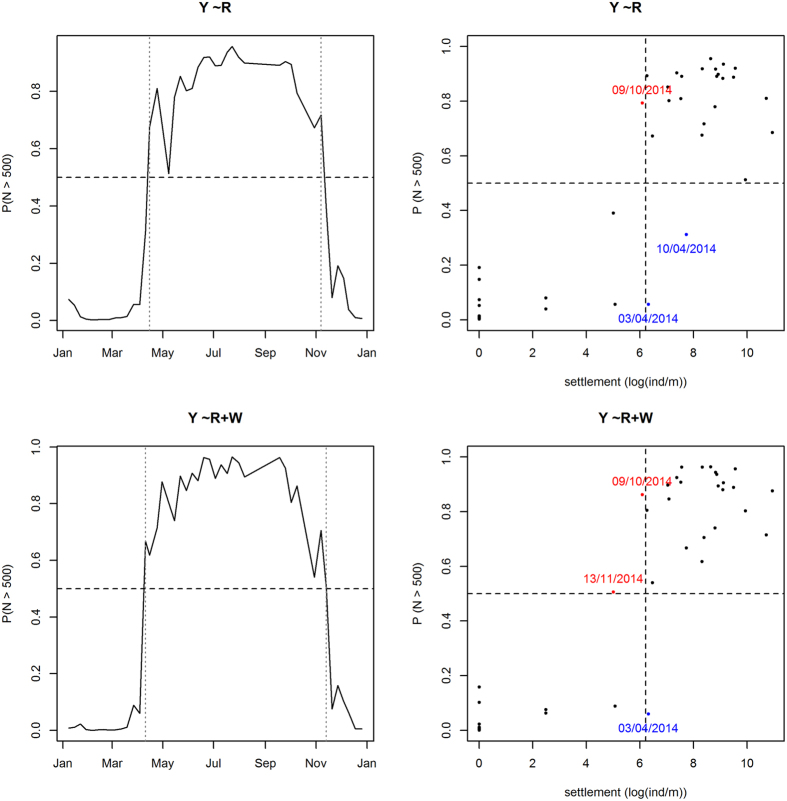
Predictions for 2014 from models fitted in the period 2009–2013. Generalized Additive Model (GAM) according to solar irradiance (top). GAM according to solar irradiance and wind regime (bottom). Left: probabilities of larval settlement along 2014, the grey dotted lines identify the beginning and end of the settlement period. Right: comparisons between settlement intensity and classification in 2014. Days with settlement misclassified are highlighted in blue, and days with residual settlement misclassified are highlighted in red.

**Figure 4:**
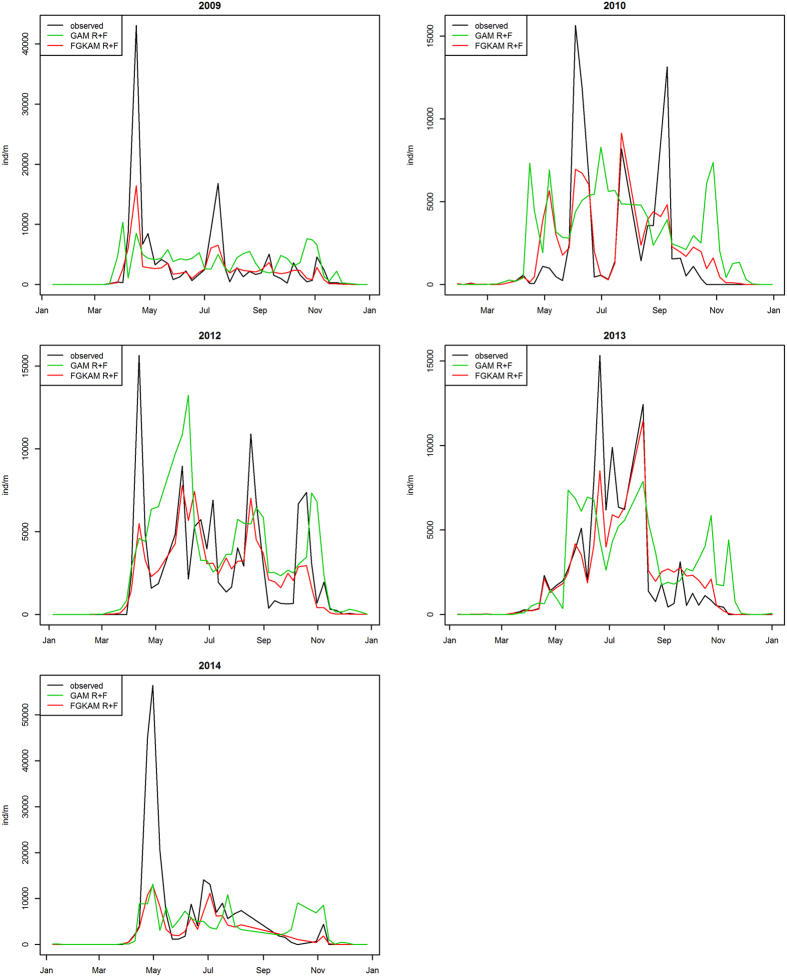
Observed settlement intensity (*N* (ind/m), black) and fitted values obtained with the GAM (green line) and FGKAM (red line) fits using solar irradiance (*R*) and continental (*Q*) as explanatory variables.

Settlement of *M. galloprovincialis* spat was monitored weekly over the periods 2009–2010 and 2012–2014 in Arnela, a cultivation area located in the southern-inner shore of the Ría de Ares-Betanzos (Fig. 1). Three ropes covered with *Scotch*-*Brite*^©^ scouring pads were deployed in the water during a week. Prior to their deployment in the field, collecting ropes were kept for 30 days in seawater filtered through a 100 μm mesh, renewing the water every 2 days to allow the development of an adequate biofilm but preventing the attachment of epifauna^31^,^43^. Sampling consisted on the collection of three sub-samples of known area (6 cm × 2 cm) from the scouring pads covering each rope at two depths (1 and 6 m), i.e. we have 18 sub-samples by sampling date. Samples were preserved in 70% ethanol until their processing in the laboratory. Sample processing consisted of the detachment of settled individuals using a 20% bleach dilution^44^, and a 5-minutes ultrasound bath. Detached individuals were then sorted using a sieve kit with mesh sizes ranging from 125 to 2360 μm, to ease their counting under a binocular microscope. The average size of individuals retained was calculated measuring the length (L, mm) of their ante-posterior axis (subsample of 100–150 individuals for large samples) for each replicate and sieve size. Settlement intensity (*N*) was calculated as the number of individuals per meter of rope (ind/m). In view of these values we set that we have significant settlement when *N* > 500 and residual settlement when *N* < 500.

### Environmental factors

The link between solar irradiance and hydrographic conditions such as sea surface temperature and food availability^14^, and the well-known effect of sea water temperature and food availability on larval production and settlement of marine invertebrates^45^,^46^,^47^ suggested using solar irradiance as explanatory variable for larval settlement. The global solar irradiance (MJ/(m^2^day)) was recorded by the Galician Meteorological Agency (Meteogalicia, http://www2.meteogalicia.es) at the neighbour meteorological station CIS-Ferrol (Fig. 1, longitude: 56078 UTMX-29T ED50, latitude: 4815885 UTMY-29T ED-50, altitude = 37 m) with a pyranometer Schenk 8101.

Shelf winds were obtained at 6 hours intervals from the Seawatch buoy of the Spanish Agency Puertos del Estado off Cape Vilano (http://www.puertos.es, Fig. 1). Gaps of less than 24 hours were interpolated linearly. For gaps of more than 24 hours, the time series was reconstructed from FNMOC model data obtained in the nearest location available (off Cape Fisterra) using generalized additive models (GAM). The goodness of fit of the GAM was around 70% of deviance explained. Reconstructed data represented 17% of the time series. Then, daily wind values were obtained by an 8^th^ order Chebyshev type I low-pass filter with cut-off frequency of 8*(Fs/2)/R, where Fs is the sampling interval and R is the rate at which data were resampled.

Continental runoff was computed summing the discharges of rivers Eume and Mandeo (Fig. 1). The flow of river Mandeo was taken from the gauge station n° 464 at Irixoa, administered by the Galician Agency Augas de Galicia. The Horton’s Law^48^ was applied to estimate the flow at the river mouth (total drainage basin: 456.97 km^2^) from the flow at the gauge station (gauged drainage basin: 248.21 km^2^). The flow of the river Eume is a combination of regulated and natural flows. Daily volumes of the Eume reservoir, which controls 80% of its drainage basin, were provided by the managing company ENDESA S.A. Assuming that the retention constant for the drainage basin of river Eume is the same than for the river Mandeo, the natural component of the flow of the river Eume was calculated again from the Horton’s Law considering the area not controlled by the reservoir (96.04 km^2^). Both time series have a daily sampling interval.

### Statistical analysis

#### Interannual variability in the environmental factors

Generalized additive models (GAM) with interaction factor (year) by curve (week)^49^,^50^ were used to fit weekly means of solar irradiance, continental runoff, and wind speed. These models can be expressed as


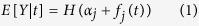


where *Y* is the response variable (solar irradiance, continental runoff or wind speed), *t* represents the week; *α*_*j*_ is the intercept for the *j*th year, and *f*_*j*_ is the unknown smooth function describing the effect of time (week) on the response for the *j*th year, which were represented using cyclic cubic penalized splines (CCRS). We used a Gamma family with logarithmic link function (*H*) to fit solar irradiance and continental runoff, which were not normal distributed (Shapiro- Wilk test: p-value < 0.001), and a Gaussian family with identity link (*H*) for wind speed. These fits allowed us to visualize the seasonal patterns of the environmental factors, and to test for interannual variability using shrinkage variable selection^51^.

#### Models for larval settlement

Model fitting of larval settlement from the environmental factors outlined above was developed in two stages: (i) predict the presence or absence of larval settlement (*Y* = *1* if *N* ≥ *500* ind/m, *Y* = *0* if *N* < *500* ind/m), (ii) predict settlement intensity (N). For both steps we considered solar irradiance (*R*, MJ/(m^2^day)), continental runoff (*Q*, m^3^/s), and wind speed (*W*, m/s^2^) and direction (θ) as explanatory variables. Generalized Additive Models (GAM)^49^,^50^ and Functional Generalized Kernel Additive Models (FGKAM)^52^ were tested to find the best approach for larval presence and settlement intensity. Prior exploratory analysis indicated that the solar irradiance occurring a month before sampling may affect more larval settlement than that occurring while the ropes are deployed. For this reason, in the GAM models, *R* represents the fortnight mean of solar irradiance recorded 30–45 days prior to sampling. On the other hand, the effect of continental runoff and wind on larval settlement is more immediate, thus to find a balance between this fact and the aim of developing a predictive model, in the GAM models *Q*, *W* and *θ* represent the mean values of the continental runoff, and shelf winds recorded between 10 and 30 days before sampling. For the functional GAM models we considered as explanatory variables the curves containing the solar irradiance (*fR*), continental runoff (*fQ*), and wind speed (*fW*) and direction (*fθ*) recorded from 15 to 45 days before sampling, i.e. up to a week prior to deploy the collector ropes.

### Generalized Additive Models (GAM)

The probability of larval settlement, *Y* = P(*N* > 500), was estimated by generalized additive models with high order interactions^53^, using the binomial family with logit link function. The shrinkage procedure^51^ provided four candidate models


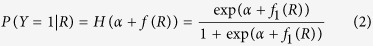



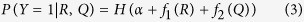



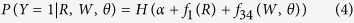



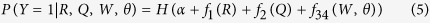


where α is the intercept, *f*_*1*_, *f*_*2*_are the unknown smooth functions for solar irradiance and continental runoff, which were represented by thin plate penalized regression splines (TPRS), and *f*_*34*_ is the smooth function for the interactions between wind speed and direction, represented as a tensor product of TPRS and CCRS estimated using scale-invariant tensor product smoothers^53^.

Settlement intensity (*N*) was fitted by a two-stage zero-inflated Poisson model, which (i) models the association between the presence-absence of larvae (*Y*) and the environmental factors, and (ii) models the relationship between settlement intensity (*N*) and the environmental factors, conditional on the presence of larvae (*Y *= 1). For this purpose we applied GAM with ziplss family, which uses a logit model in (i) to estimate the presence of larvae, and a Poisson model with identity link in (ii) to fit settlement intensity. The shrinkage variable selection^51^ suggested the following candidate models.


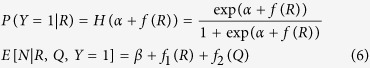



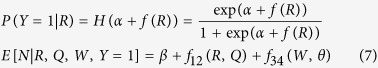


where, α and β are the intercepts of the first and second stages, respectively. The smooth terms for solar irradiance, *f*_*1*_, and continental runoff, *f*_*2*_, were represented by TPRS, the smooth function accounting for interactions between solar irradiance and continental runoff, *f*_*12*_, was represented as a tensor product of TPRS, and the interaction between wind speed and direction, *f*_*34*_, was represented by a tensor product of TPRS and CCRS. Both *f*_*12*_ and *f*_*34*_ were estimated using scale-invariant tensor product smoothers^53^.

### Functional Generalized Kernel Additive Models (FGKAM)

In functional data analysis^35^ (FDA) we assume that a high dimensional vector represents a set of discrete observations of a continuous function. This method replaces the sampled functions (discrete observations) by functional representations (curves). In this work, the functional data are curves measuring the evolution of solar irradiance (*fR*) continental runoff (*fQ*) and wind speed (*fW*) during a month.

Functional data that account for the evolution of the environmental factors recorded between 15 and 45 days prior to samplings were considered as explanatory variables, to estimate settlement intensity (*N*) by Functional Generalized Kernel Additive Models^52^ with negative binominal family, as we are dealing with over-dispersed counting data. We evaluated the following models,


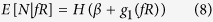



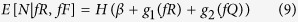


where β is a functional parameter, *g*_*1*_ and *g*_*2*_are the unknown smooth functions for solar irradiance and continental runoff, and *H* is the logarithmic link function. The smooth functions in models (8)-(9) were estimated applying Nadaraya-Watson weighted kernel smoothers^52^.

### Model selection

The goodness-of-fit of the models outlined above can be measured in terms of probability of correct classification for larval absence-presence (*Y*) and by global error measures such as the Mean Square Error (MSE) or the Mean Absolute Error (MAE) for settlement intensity (*N*).

We applied the following algorithm in order to select the best model to predict larval occurrence and settlement intensity among the candidate models proposed above:

1. Use simple random sampling to split the dataset into a training sample, containing 75% of the data, and a validation sample containing the remaining 25%.
2. Use the training sample to fit each model.
  - Obtain the probability of correct classification for models (2)–(5).
  - Obtain the MSE and MAE of the residuals for models (6)–(9).
3. Use the fitted models to predict the response in the validation sample
  - Obtain the probability of correct classification for models (2)–(5).
  - Obtain the MSE and MAE of the residuals for models (6)–(9).
4. Repeat steps (i)–(iii) 1000 times.

## Additional Information

**How to cite this article**: Fuentes-Santos, I. *et al*. Solar irradiance dictates settlement timing and intensity of marine mussels. *Sci. Rep.* **6**, 29405; doi: 10.1038/srep29405 (2016).

## Supplementary Material

Supplementary Information

## Figures and Tables

**Table 1:** Proportion of correct classifications (mean and standard deviations) for the GAM models (2) to (5) in the 1000 training and validation samples.

Training sample					Validation sample		
		Correct	N < 500	N ≥ 500	Correct	N < 500	N ≥ 500
*R*	Mean	0.876	0.828	0.918	0.872	0.825	0.914
	Sd	0.0139	0.0262	0.0135	0.0373	0.0695	0.0489
*R* + *Q*	Mean	0.875	0.829	0.917	0.868	0.822	0.910
	Sd	0.0144	0.0264	0.0141	0.0381	0.0694	0.0517
*R* + W*θ	Mean	0.915	0.865	0.960	0.885	0.835	0.931
	Sd	0.0196	0.0327	0.0132	0.0409	0.0648	0.0551
*R* + *Q* + W*θ	Mean	0.920	0.873	0.962	0.881	0.834	0.923
	sd	0.0257	0.0420	0.0154	0.0439	0.0646	0.0626

*R* (2): GAM fit according to the mean solar irradiance recorded the 30–45 days before sampling. *R* + *Q* (3): GAM fit according to *R* and *Q* (mean continental runoff 10–30 days before sampling), *R* + W*θ (4): GAM model according to *R* and W*θ (joint effect of the mean wind speed and direction 10–30 days before sampling). *R* + *Q* + W*θ (5): GAM fit according to solar irradiance, continental runoff, and wind regime. The GAM fits were conducted with the binomial family.

**Table 2:** One-side Wilcoxon test for comparison of probabilities of correct classification among the GAM models.

		Correct classification			Correct classif N < 500			Correct classif N > 500		
		*R* + *F*	*R* + W*θ	*R* + *Q* + W*θ	*R* + *Q*	*R* + W*θ	*R* + *Q* + W*θ	*R* + *Q*	*R* + W*θ	*R* + *Q* + W*θ
Train	*R*	1	<2e-16	<2e-16	0.2823	<2e-16	<2e-16	1	<2e-16	<2e-16
	*R* + *Q*	0	<2e-16	<2e-16	0	<2e-16	<2e-16	0	<2e-16	<2e-16
	*R* + W*θ	0	0	<2e-16	0	0	<2e-16	0	0	<2e-16
Validate	*R*	1	<2e-16	<2e-16	1	<2e-16	<2e-16	1	<2e-16	<2e-16
	*R* + *Q*	0	<2e-16	<2e-16	0	<2e-16	<2e-16	0	<2e-16	<2e-16
	*R* + W*θ	0	0	1	0	0	0.9700	0	0	1

See details in the legend of Fig. 1.
