# Supplementary material for: Solar irradiance dictates settlement timing and intensity of marine mussels
Source: Sci Rep. 2016 Jul 7;6:29405. doi: 10.1038/srep29405 (PMC4935941; doi:10.1038/srep29405)
Supplement: Supplementary Information [file srep29405-s1.pdf]

SUPPLEMENTARY INFORMATION FOR

Solar irradiance dictates settlement timing and intensity of marine mussels

Isabel Fuentes-Santos, Uxío Labarta*, X. Antón Álvarez-Salgado, Mª José Fernández-Reiriz

Consejo Superior de Investigaciones Científicas (CSIC), Instituto de Investigaciones Marinas (IIM), C/Eduardo Cabello 6, 36208 Vigo, Spain

*Corresponding author: labarta@iim.csic.es

Telephone: +34 986 231 930 Ext: 214.

FAX:(+34) 986 292 762

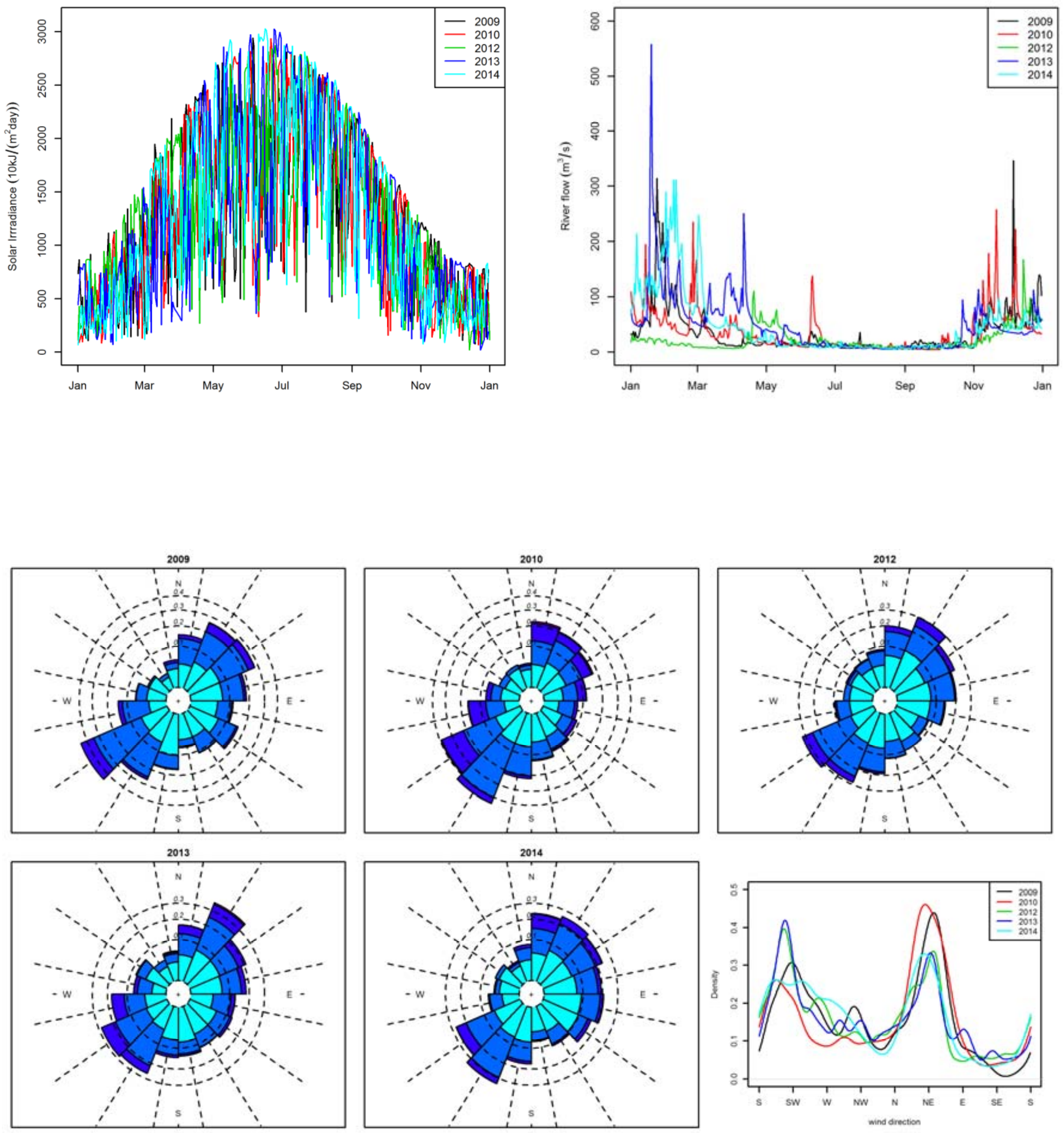

Figure S1: Observed values of solar irradiance (top, left), continental runoff (top, right) and wind stress (bottom, windroses and circular density estimator for wind direction by years.

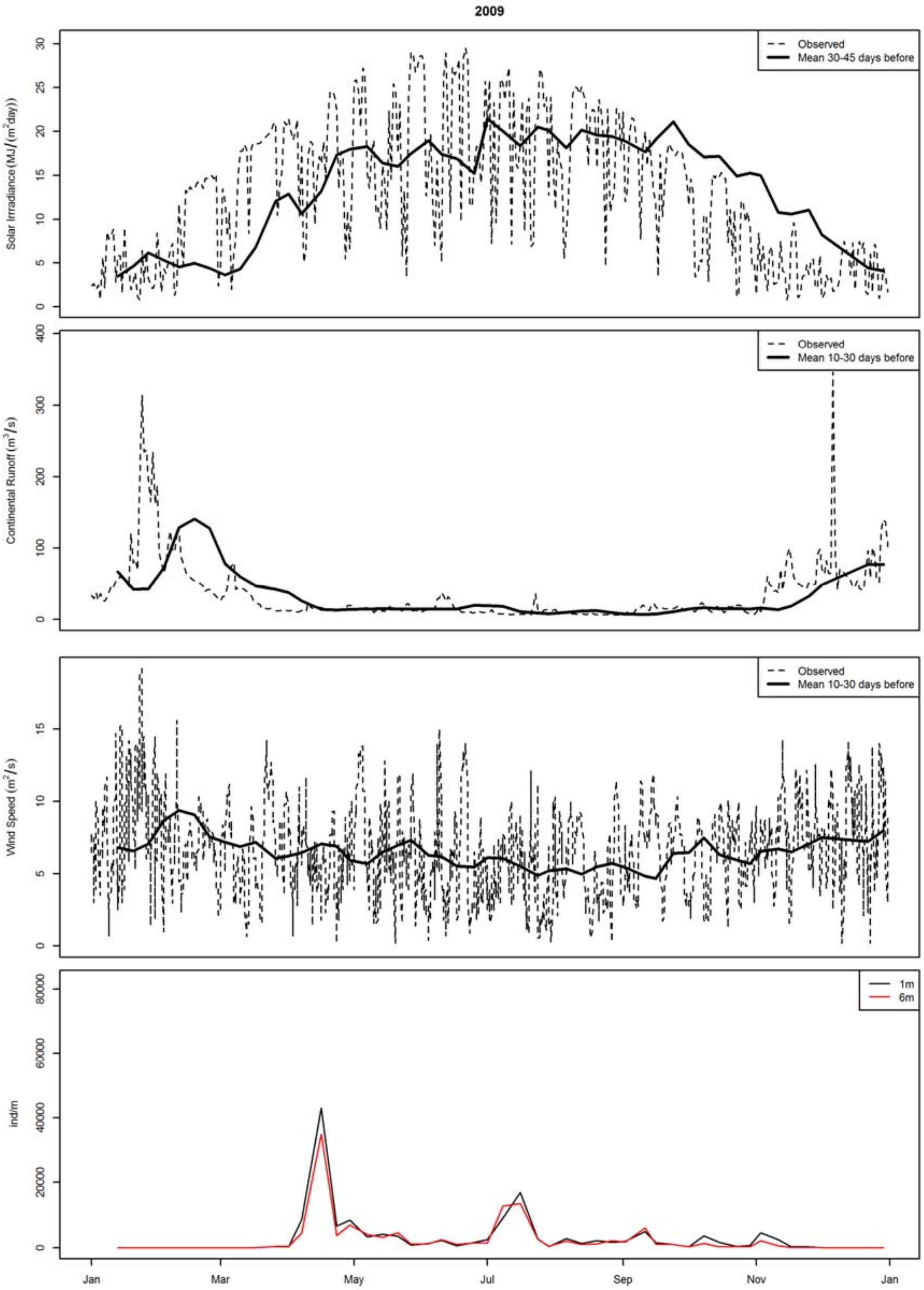

Figure S2: Environmental conditions and larval settlement during 2009. Solar irradiance: daily values (dashed line) and fortnightly means 30-45 days prior to sampling (solid line). Continental runoff and wind speed: daily values (dashed lines) and mean of the values recorded 10-30 days prior to sampling (solid lines). Settlement intensity at 1 and 6m.

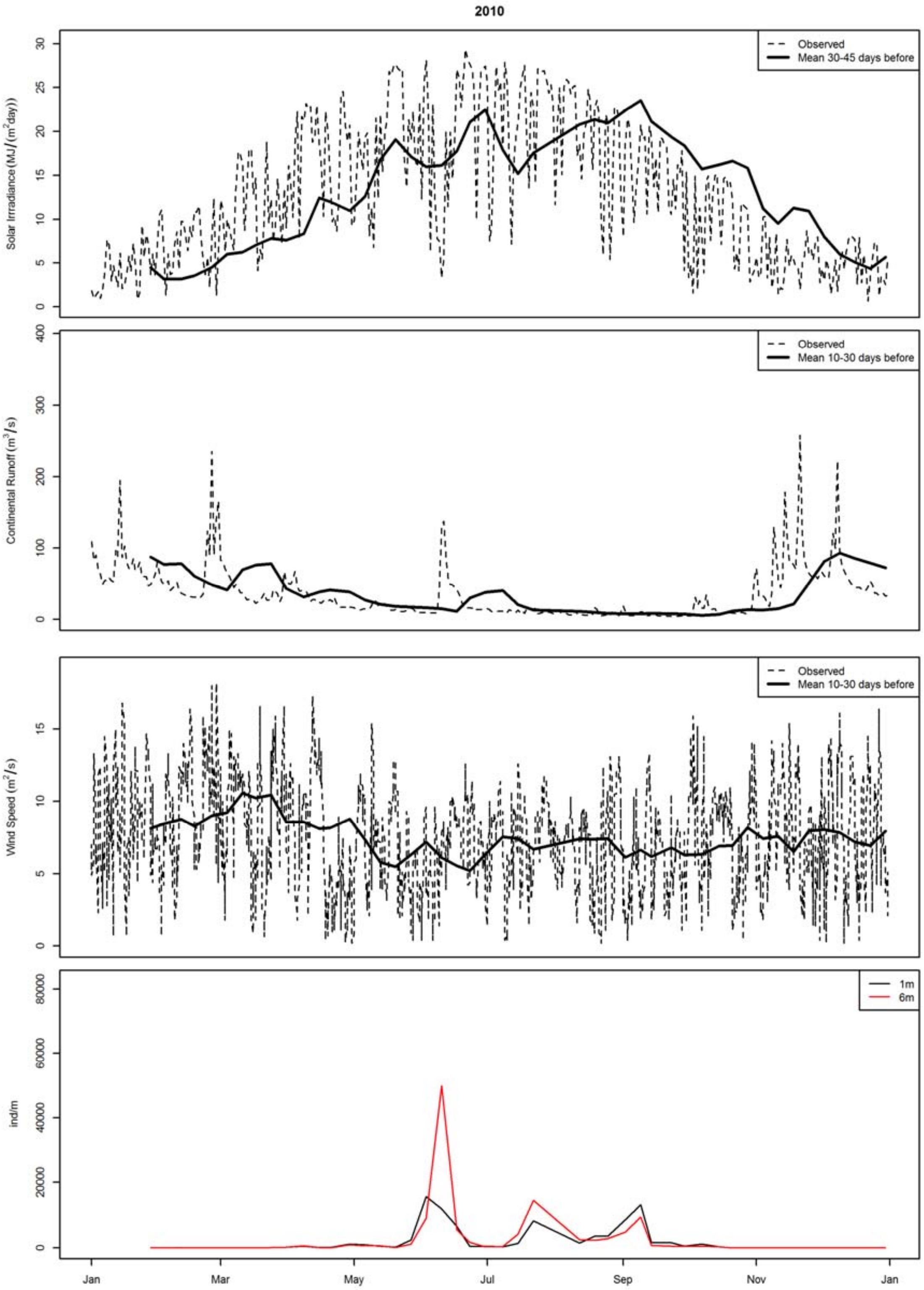

Figure S3: Environmental conditions and larval settlement during 2010. Solar irradiance: daily values (dashed line) and fortnightly means 30-45 days prior to sampling (solid line). Continental runoff and wind speed: daily values (dashed lines) and mean of the values recorded 10-30 days prior to sampling (solid lines). Settlement intensity at 1 and 6m.

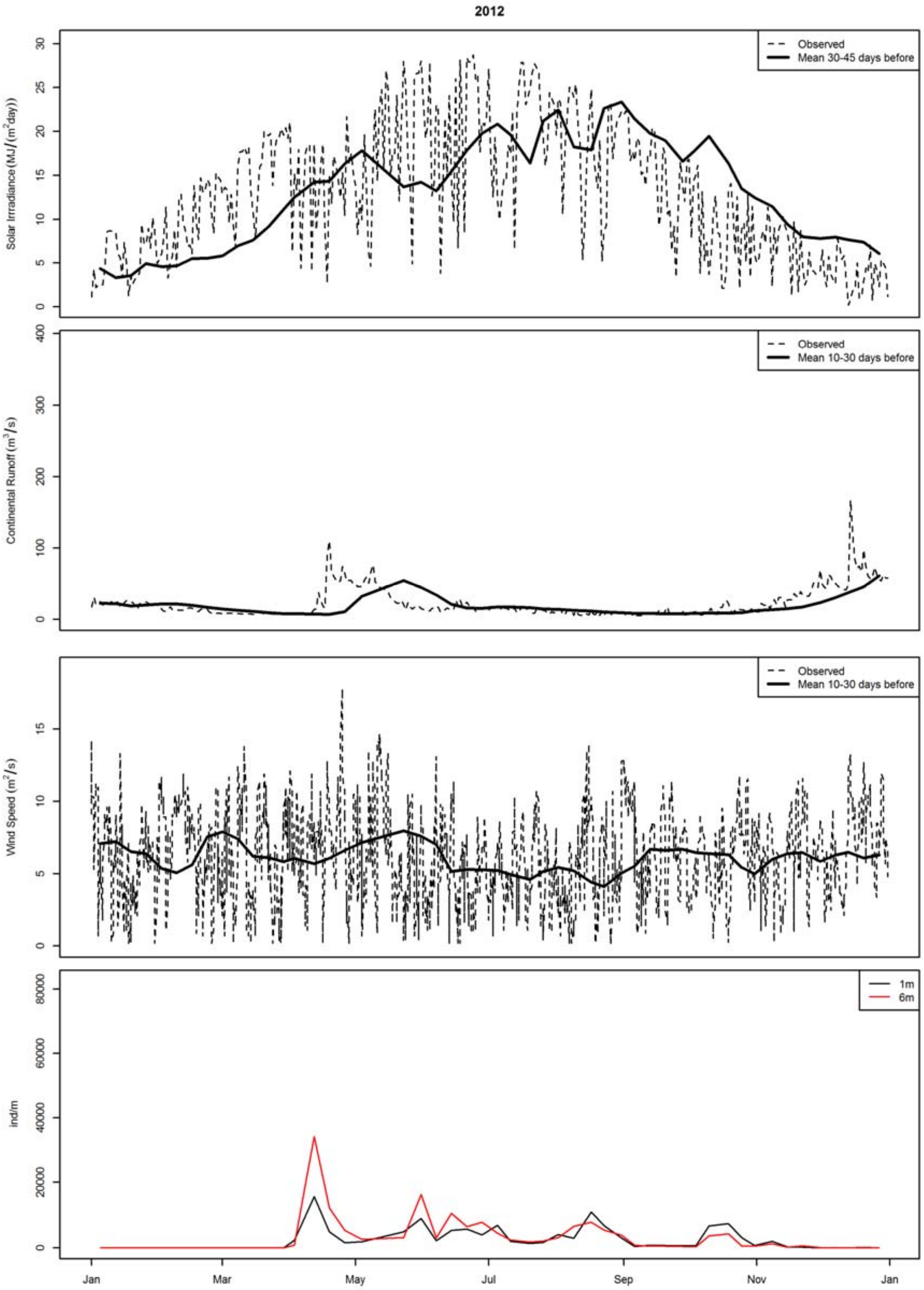

Figure S4: Environmental conditions and larval settlement during 2012. Solar irradiance: daily values (dashed line) and fortnightly means 30-45 days prior to sampling (solid line). Continental runoff and wind speed: daily values (dashed lines) and mean of the values recorded 10-30 days prior to sampling (solid lines). Settlement intensity at 1 and 6m.

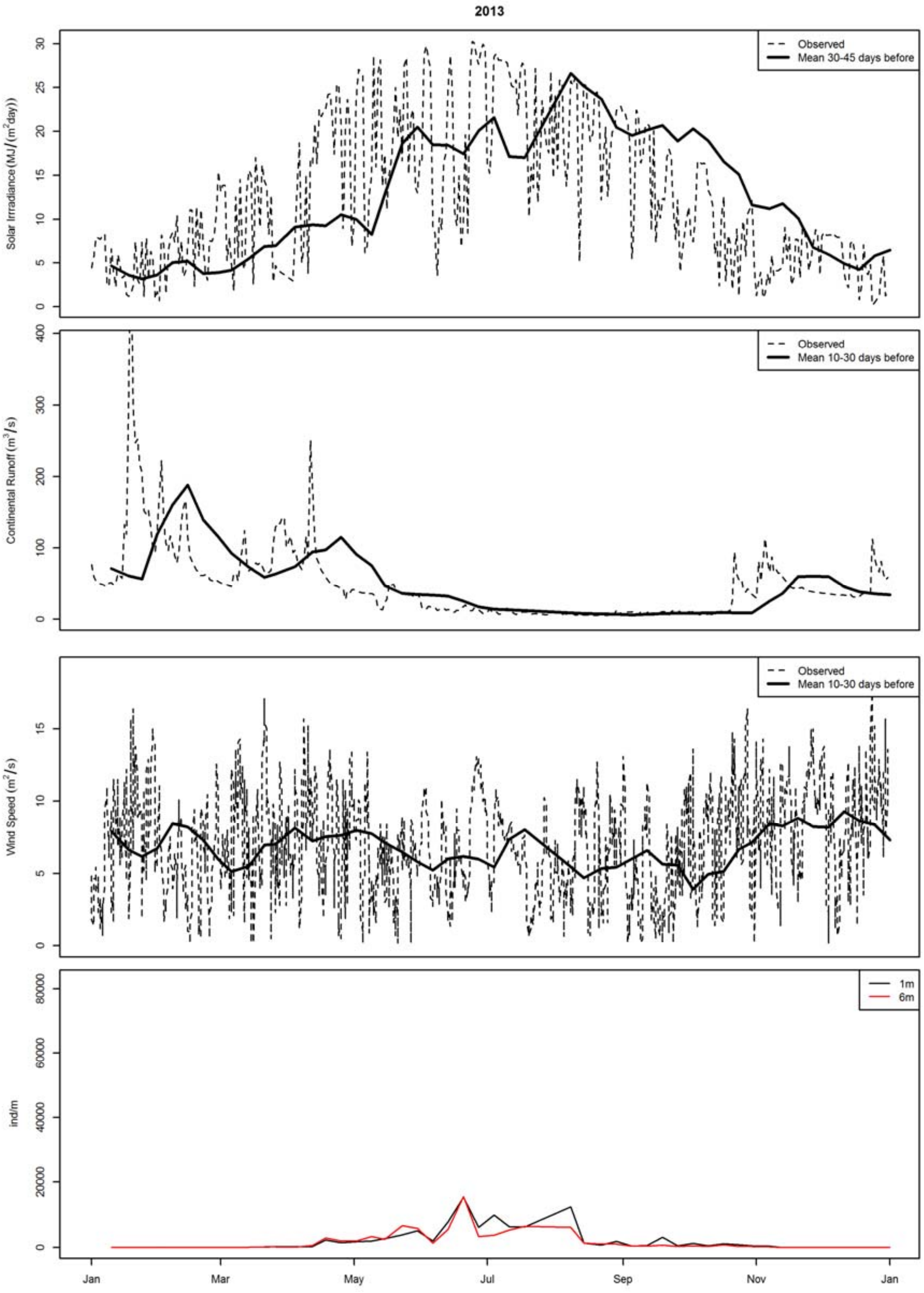

Figure S5: Environmental conditions and larval settlement during 2013. Solar irradiance: daily values (dashed line) and fortnightly means 30-45 days prior to sampling (solid line). Continental runoff and wind speed: daily values (dashed lines) and mean of the values recorded 10-30 days prior to sampling (solid lines). Settlement intensity at 1 and 6m.

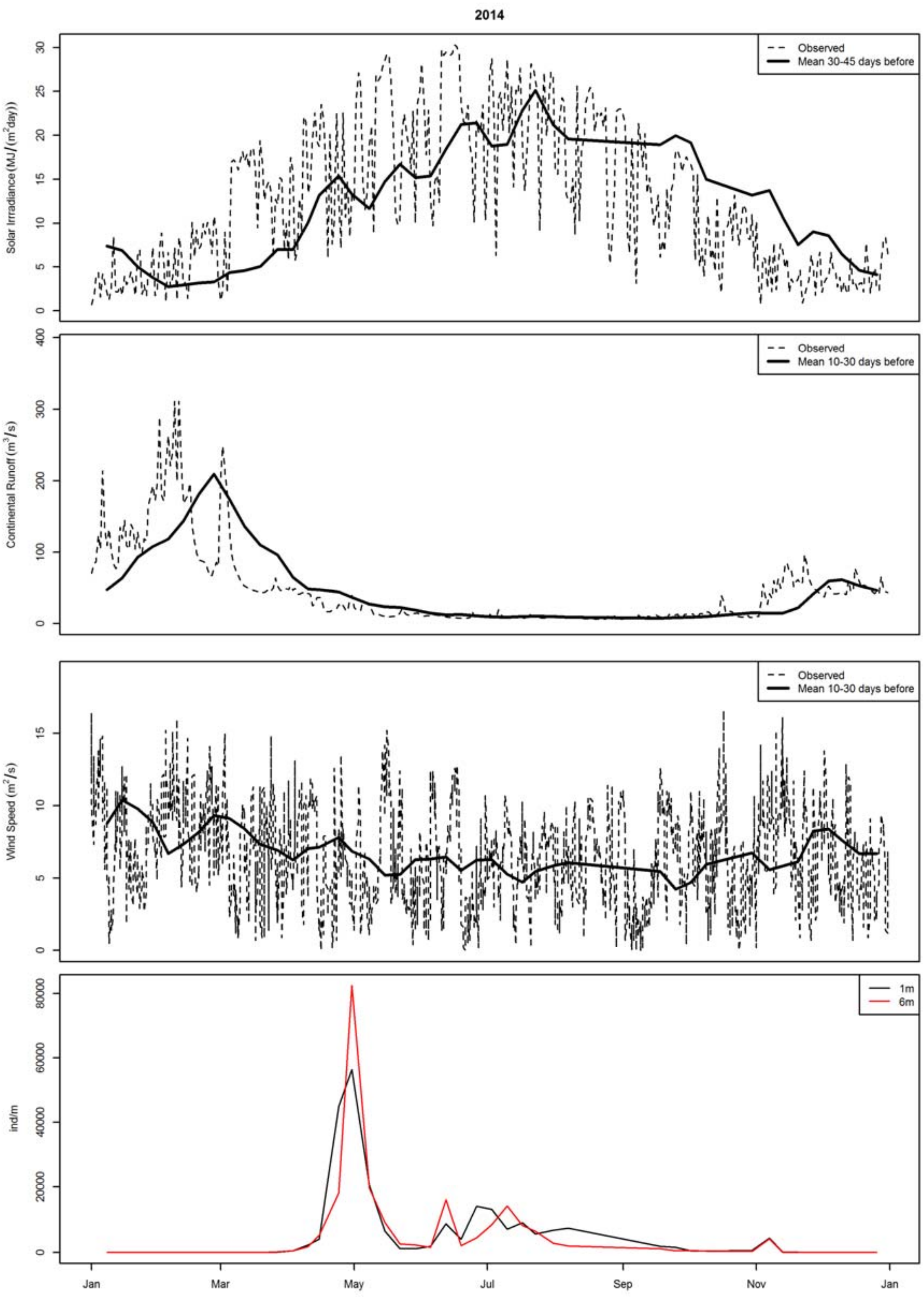

Figure S6: Environmental conditions and larval settlement during 2014. Solar irradiance: daily values (dashed line) and fortnightly means 30-45 days prior to sampling (solid line). Continental runoff and wind speed: daily values (dashed lines) and mean of the values recorded 10-30 days prior to sampling (solid lines). Settlement intensity at 1 and 6 m.

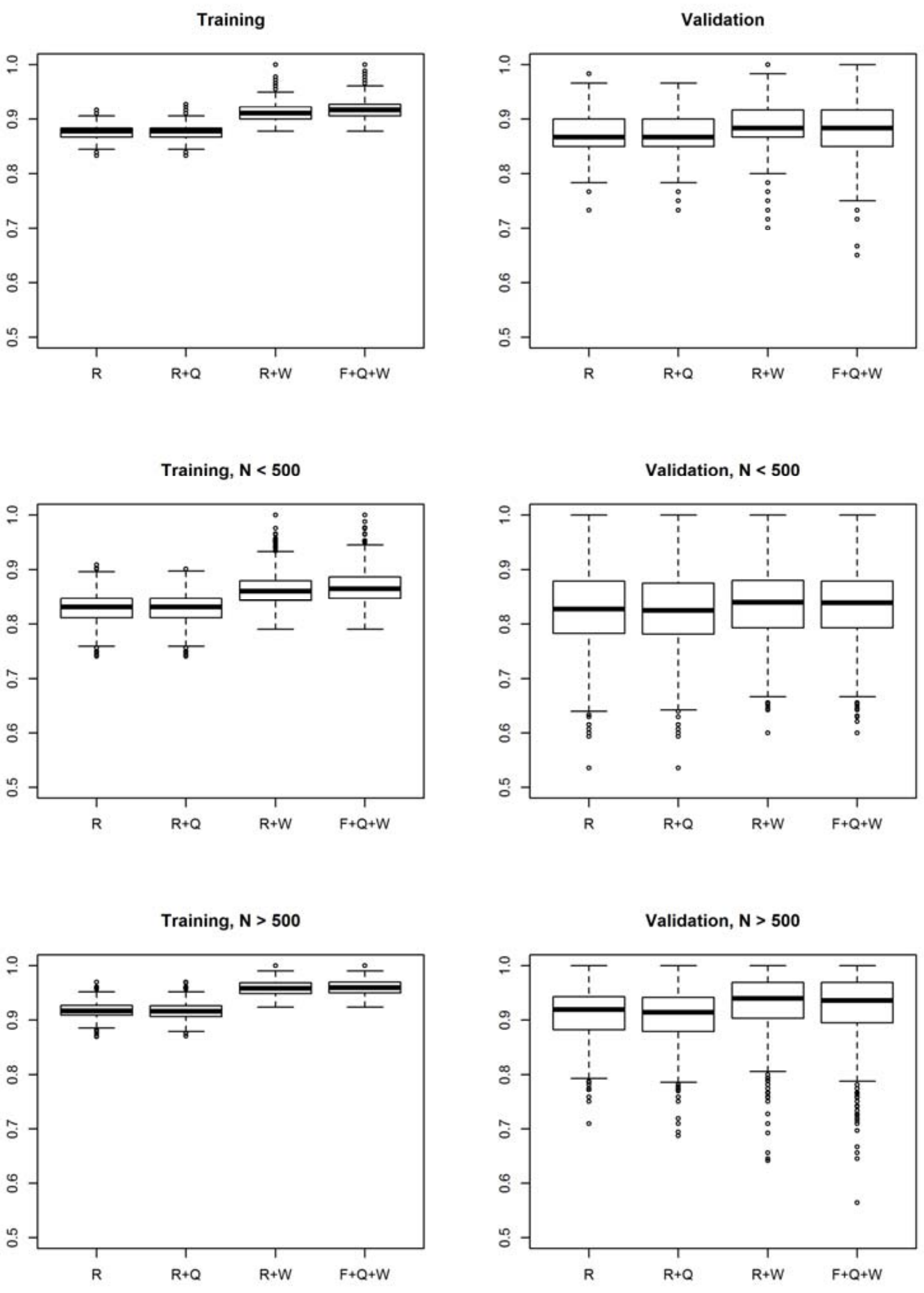

Figure S7: Probability of correct classification (top), correct identification of residual larval intensity ($N < 500$, centre) and significant larval settlement ($N \geq 500$, bottom) for 1000 training (left, 75% of data) and validation samples (right, 25% of data). *R*: GAM fit according to the mean solar irradiance registered 30-45 days before sampling. *R+ Q*: GAM fit according to R and mean continental runoff of 10-30 days before sampling (*Q*), *R+ W*: GAM model according to *R* and the interaction between the mean wind speed and direction registered 10-30 days before sampling (*W*). *R+Q+W*: GAM fit according to solar irradiance (*R*), continental runoff and wind regime (*W*). GAM fits were conducted with the binomial family.

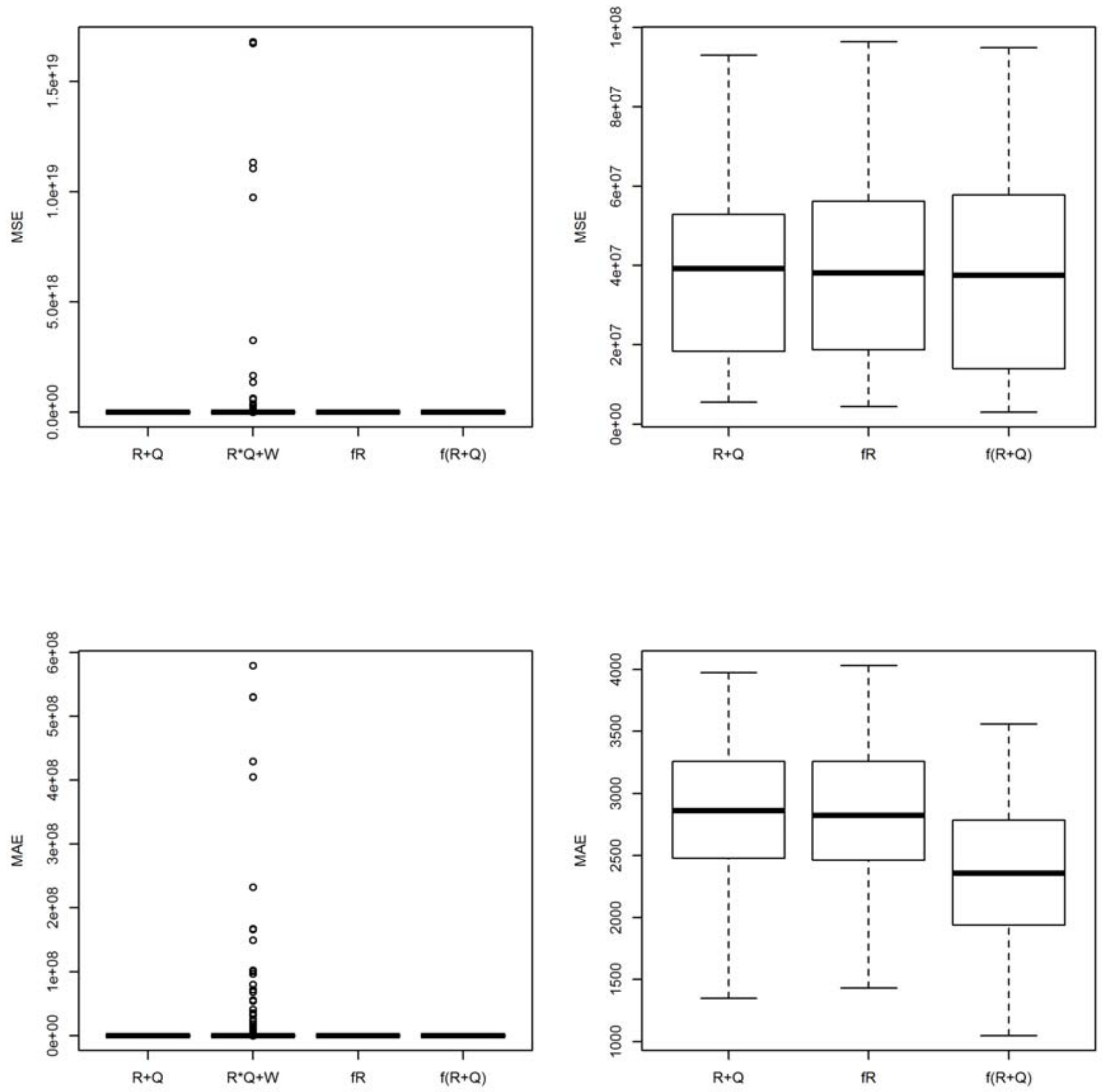

Figure S8: Goodness-of-fit of the different models tested measured in terms of MSE (mean squared error, top), and MAE (mean absolute error, bottom) for the 1000 validation samples. *R+Q*: GAM fit according to the mean solar irradiance recorded 30-45 days before sampling (*R*) and mean continental runoff 10-30 days before sampling (*Q*). R*Q+W: GAM fit according to the interaction of *R*, *Q* and the interaction between the mean wind speed and direction recorded 10-30 days before sampling (*W*). The GAM models were fitted with the ziplss family considering *R* as explanatory variable to estimate the probability of significant settlement. *fR*: functional GAM fit according to the curves of solar irradiance from 15 to 45 days before sampling. *f(R+Q)*: functional GAM fit according to the curves of solar irradiance and continental runoff from 15 to 45 days prior to sampling.

Table S1: Results of the model selection procedure conducted to test for the effect of year on the seasonal patterns of solar irradiance, continental runoff and wind speed.

Model Fitting								
		Parametric coefficients					Goodness-of-fit	
		Estimate	Std. Error	t value	Pr(>|t|)		Adj R^2	%Dev Exp
**Solar irradiance**	2009	6.992	0.036	194.1	<2e-16	***	0.831	0.835
	2010	-0.006	0.051	-0.126	0.9			
	2012	0.002	0.051	0.041	0.967			
	2013	-0.022	0.051	-0.434	0.665			
	2014	-0.038	0.051	-0.747	0.456			
		Smoth terms						
		edf	Ref.df	F	p-value			
	s(week)	5.39	8	108.333	<2e-16	***		
	s(week):2009	3.34	8	0.954	0.0469	*		
	s(week):2010	0.00	8	0	0.9115			
	s(week):2012	5.67	8	1.644	0.0219	*		
	s(week):2013	0.00	8	0	0.8085			
	s(week):2014	0.13	8	0.022	0.2633			
		Parametric coefficients					Goodness-of-fit	
		Estimate	Std. Error	t value	Pr(>|t|)		Adj R^2	%Dev Exp
**Continental runoff**	2009	3.215	0.053	60.583	<2e-16	***	0.831	0.835
	2010	0.091	0.075	1.215	0.2256			
	2012	-0.324	0.075	-4.337	2.2e-07	***		
	2013	0.269	0.075	3.59	0.0004	***		
	2014	0.103	0.075	1.372	0.1714			
		Smoth terms						
		edf	Ref.df	F	p-value			
	s(week)	7.70	8	44.967	<2e-16	***		
	s(week):2009	4.88	8	3.506	8.0e-06	***		
	s(week):2010	6.93	8	4.97	5.1e-07	***		
	s(week):2012	7.13	8	22.803	<2e-16	***		
	s(week):2013	7.16	8	3.395	0.0002	***		
	s(week):2014	0.00	8	0	0.0063	**		
		Parametric coefficients					Goodness-of-fit	
		Estimate	Std. Error	t value	Pr(>|t|)		Adj R^2	%Dev Exp
**Wind speed**	2009	6.59	0.21934	30.059	<2e-16	***	0.224	26.80%
	2010	0.90	0.3102	2.915	0.0039	**		
	2012	-0.37	0.30878	-1.196	0.2328			
	2013	0.37	0.3102	1.205	0.2294			
	2014	-0.02	0.3102	-0.073	0.9421			
		Smoth terms						
		edf	Ref.df	F	p-value			
	s(week)	2.34	8	3.264	2.8e-07	***		
	s(week):2009	0.00	8	0	0.2456			
	s(week):2010	3.64	8	0.525	0.2983			
	s(week):2012	0.00	8	0	0.8764			
	s(week):2013	5.20	8	1.219	0.0668	.		
	s(week):2014	0.00	8	0	0.7052			

Table S2: Minimum irradiance level needed to obtain settlement probabilities higher than 0.5 (I), 0.75 (II) and 0.9 (III) for the GAM fit according to solar irradiance. Probability of correct classification with GAM fits (2) and (4): proportion of weeks with ($N \geq 500$) and without ($N <$ 500) settlement correctly classified. Irradiance threshold for each year and percentages of correct classification were obtained by cross-validation, i.e. settlement probabilities at year *j* were obtained fitting the models in the remainder years.

Year	Model	IRRADIANCE THERSHOLD			% CORRECT CLASSIFICATION	
		I	II	III	N < 500	N ≥ 500
2009-2014	*Y~R*	11.46	14.23	18.49	84.07%	92.13%
	*Y~R+W*θ*				84.96%	95.28%
2009	*Y~R*	12.06	14.92	18.9	75.00%	93.10%
	*Y~R+W*θ*				75.00%	96.55%
2010	*Y~R*	10.93	15.18	15.72	69.57%	94.74%
	*Y~R+W*θ*				72.00%	94.74%
2012	*Y~R*	12.47	15.46	19.45	95.24%	96.55%
	*Y~R+W*θ*				95.24%	82.76%
2013	*Y~R*	11.63	15.12	19.55	91.67%	84.62%
	*Y~R+W*θ*				91.67%	88.46%
2014	*Y~R*	11.68	14.73	19.96	95.00%	91.67%
	*Y~R+W*θ*				90.00%	95.83%
